# Human Probing Behavior of *Aedes aegypti* when Infected with a Life-Shortening Strain of *Wolbachia*

**DOI:** 10.1371/journal.pntd.0000568

**Published:** 2009-12-15

**Authors:** Luciano A. Moreira, Emad Saig, Andrew P. Turley, José M. C. Ribeiro, Scott L. O'Neill, Elizabeth A. McGraw

**Affiliations:** 1 School of Biological Sciences, The University of Queensland, St. Lucia, Queensland, Australia; 2 Laboratory of Malaria, Rene Rachou Research Center, CPqRR-FIOCRUZ, Belo Horizonte, Minas Gerais, Brazil; 3 Laboratory of Malaria and Vector Research, National Institute of Allergy and Infectious Diseases, National Institutes of Health, Rockville, Maryland, United States of America; National Yang-Ming University, Taiwan

## Abstract

**Background:**

Mosquitoes are vectors of many serious pathogens in tropical and sub-tropical countries. Current control strategies almost entirely rely upon insecticides, which increasingly face the problems of high cost, increasing mosquito resistance and negative effects on non-target organisms. Alternative strategies include the proposed use of inherited life-shortening agents, such as the *Wolbachia* bacterium. By shortening mosquito vector lifespan, *Wolbachia* could potentially reduce the vectorial capacity of mosquito populations. We have recently been able to stably transinfect *Aedes aegypti* mosquitoes with the life-shortening *Wolbachia* strain *w*MelPop, and are assessing various aspects of its interaction with the mosquito host to determine its likely impact on pathogen transmission as well as its potential ability to invade *A. aegypti* populations.

**Methodology/Principal Findings:**

Here we have examined the probing behavior of *Wolbachia*-infected mosquitoes in an attempt to understand both the broader impact of *Wolbachia* infection on mosquito biology and, in particular, vectorial capacity. The probing behavior of *w*MelPop-infected mosquitoes at four adult ages was examined and compared to uninfected controls during video-recorded feeding trials on a human hand. *Wolbachia*-positive insects, from 15 days of age, showed a drastic increase in the time spent pre-probing and probing relative to uninfected controls. Two other important features for blood feeding, saliva volume and apyrase content of saliva, were also studied.

**Conclusions/Significance:**

As *A. aegypti* infected with *w*MelPop age, they show increasing difficulty in completing the process of blood feeding effectively and efficiently. *Wolbachia*-infected mosquitoes on average produced smaller volumes of saliva that still contained the same amount of apyrase activity as uninfected mosquitoes. These effects on blood feeding behavior may reduce vectorial capacity and point to underlying physiological changes in *Wolbachia*-infected mosquitoes.

## Author Summary

Mosquitoes transmit diseases when they are actively searching for a source of blood. This so called probing behavior comprises the “searching” time, the beginning of the feeding process until the first sign of blood can be seen within the insect body. The manipulation of this behavior can have important consequences for the mosquito's ability to transmit pathogens, such as dengue virus or *Plasmodium*. In this study we examined the probing behavior of the main vector of dengue viruses, *Aedes aegypti*, when infected with an intracellular bacterium, *Wolbachia pipientis*. This bacterium alters the probing behavior of older mosquitoes such that they take longer to find a feeding site and longer to imbibe blood, which may make them more susceptible to human defense responses. The bacterium appears to reduce mosquito feeding success by preventing the mosquito from successfully inserting its stylet into human skin. The old age onset of reduced mosquito feeding success due to *Wolbachia* could selectively promote a reduction in dengue transmission.

## Introduction

Insect transmitted diseases such as malaria and dengue occur in more than 100 countries worldwide, placing at risk around half the world's population. The disease burden is high with more than 500 million cases each year. Despite various vector control measures, there continues to be emergence and resurgence of these diseases [1]. *Aedes aegypti* is the main vector of dengue fever causing millions of cases and thousands of deaths each year. Vector control is the only method for dengue and dengue hemorrhagic fever (DHF) prevention, however current strategies are failing to prevent the increasing global incidence of dengue fever [2]. The development of practical alternative strategies to control dengue, which could be used in conjunction with current measures, is much needed.

Recently our group reported the successful stable infection of *A. aegypti* with the *w*MelPop *Wolbachia* strain that reduces insect lifespan [3]. *Wolbachia* is an inherited bacterium able to manipulate the insect host's reproductive biology [4] in a manner that promotes its rapid spread through insect populations [5]. Releasing *Wolbachia*-infected mosquitoes that could initiate an invasion of *Wolbachia* into a wild mosquito population [6] and that resulted in reduced lifespan of wild mosquitoes could theoretically greatly reduce transmission of dengue virus. This is because only old mosquitoes transmit the virus [3],[7],[8]. In addition to lifespan reduction, we have recently shown that the *w*MelPop infection substantially reduces dengue load in *A. aegypti* mosquitoes [9] and reduces their ability to successfully obtain blood meals as they age [10].

Mosquitoes rely on chemical and physical cues (e.g. carbon dioxide, body odors, air movement and heat) to locate suitable hosts for feeding [11],[12] and this complex set of activities is known as host-seeking behavior [13],[14]. Once the host is located, the mosquito must quickly obtain blood to avoid any host defensive behavior [15],[16]. Behavior during feeding can then be divided into the stages of pre-probing (foraging) [17] and probing (feeding) activities [18]. After finding a suitable blood vessel and thrusting its stylet into the host skin [19], the saliva plays an important role in preventing blood clotting, through the anti-platelet aggregation activity of the enzyme apyrase [20],[21] and other antihemostatic and anti-inflammatory compounds [19]. Insect vector competence for the transmission of viruses and parasites is dependent upon the successful execution of these upstream steps in the process.

Here we report the results of an examination of the effects of *w*MelPop infection on pre-probing behavior, probing behavior, saliva production, and apyrase content of saliva of *A. aegypti*. The goals of the study were to identify possible mechanisms for the insect's reduced ability to obtain a blood meal with age and further evaluate the capacity for *Wolbachia* infection to reduce vector competence.

## Methods

### Ethics statement

This study was conducted according to the principles expressed in the Declaration of Helsinki. The study was approved by the Medical Research Ethics Committee at the University of Queensland (Project #2007001379). Volunteers were made aware of the risks of bloodfeeding (allergy and discomfort) and the plans to analyze and publish all data prior to providing written consent to participate in the study.

### Mosquitoes

*Aedes aegypti* mosquitoes, *w*MelPop infected (PGYP1) and its Tetracycline-cured counterpart (PGYP1.tet) [3], were kept in a controlled environment insectary at 25 °C and 80% RH. Larvae were maintained with fish food pellets (Tetramin, Tetra) and adults were offered 10% sucrose solution, *ad libitum*. Adult females were fed on human blood (UQ human ethics approval 2007001379) for egg production and eggs were dried for at least 96 h prior to hatching.

### Behavior assays

Fertilized and non-blood fed females of different ages (5, 15, 26 and 35 days old) were used in all behavior experiments. Sucrose solutions were removed from cages on the night before the experiments. Forty females were used per age and per infectious status. Single mosquitoes were transferred to a transparent Perspex cage (25 cm^3^) and filmed through a digital camera with 6mm Microlens (IEEE-1394, Point Grey Research) mounted on a tripod. Mosquitoes were given about five minutes to settle within the cage before a human gloved-hand was inserted into the cage. A window of about 15 cm^2^ was cut of the upper part of the latex glove in order to delineate the probing field.

Movies were recorded (QuickTime Player) for a maximum of 10 minutes or until blood was seen within the mosquito midgut and sub-sequentially watched for time calculations. Two electronic timers were used, one for recording pre-probing time and the second for probing time. Pre-probing time was defined and the time since the mosquito has landed on the bare hand area until the insertion of mouthparts into the human skin. Probing time is defined as the initial insertion of insect mouthparts until blood can be seen within the mosquito midgut through the abdominal pleura [21]. Timing stopped when mosquitoes left the bare hand area or withdrew their mouthparts before taking blood and began again when the mosquito came back or after subsequent stylet penetration. If blood was not found by the end of 10 minutes, we defined this case as unsuccessful probing and it was measured as a proportion. Movies were also used to visualize additional abnormal phenotypes as the jittering action of mosquito body while landed on top of the human hand, and named “shaky”. Furthermore, the inability of mosquitoes to insert they mouthparts due to a bendy proboscis was also analyzed. The bendy phenotype was recently described by Turley et al [10].

### Mosquito saliva collection

Mosquitoes of different ages (5, 26 and 35-days-old) and infectious status were starved overnight (without sucrose solution or water). On the following morning mosquitoes were briefly anesthetized with CO_2_ and placed on a glass plate over ice. Wings and legs were removed with forceps and their proboscis introduced into a 1cm piece of polypropylene tubing (0.61×0.28mm, Microtube Extrusions, NSW, Australia) (modified from [21]). Females were allowed to salivate for 5 minutes and then the diameter of the saliva droplets was measured through an ocular micrometer at 40× magnification. Volumes were calculated via the sphere formula [22]. Saliva was then collected into 20 µL of 0.05mM Tris-HCl pH 7.5 by attaching the needle of a 10 µL Hamilton syringe and rinsing the tubing content a few times. Samples were centrifuged at 14,000g for 2 minutes and kept frozen (−80°C) in 20 µL of 0.05mM Tris-HCl, pH 7.5 for enzymatic assay (see below).

### Apyrase assay

Saliva samples (8 µL) were transferred, in duplicates, into individual wells of a plastic 96-well ELISA plate (NUNC Maxisorp). For the blank, 8 µL of the 0.05mM Tris buffer was added to the wells. To each well, 100 µL was added of a mixture containing 100mM NaCl, 50mM Tris–HCl (pH 8.95), 5mM CaCl_2_, 2mM ATP and 20 mM B-Mercapthanol. The plate was incubated at 37°C for 10 min and then the reaction was immediately stopped, by adding 25 uL of acid molybdate solution (1.25% ammonium molybdate in 2.5mM H_2_SO_4_). Immediately after termination of the reaction, 2 µL of a reducing solution (0.11mM NaHSO_3_, 0.09mM Na_2_SO_3_ and 8mM 1-amino-2-naphthol-4-sulphonic acid) was added to each well and the plate was incubated at 37°C for 20 min [22]. Plates were read at a FLUOstar OPTIMA ELISA plate reader (BMG Technologies) at 660nm. Readings were quantified by comparison with an inorganic phosphate standard curve (1, 0.5, 0.25, 0.125, 0.06125, 0.03125, 0.015625 mM of sodium phosphate).

### PCR confirmation of mosquito infection status and saliva screening

*Wolbachia* infection was confirmed through PCR to detect both mosquito (apyrase gene: ApyF: 5′-TTTCGACGGAAGAGCTGAAT-3′ and ApyR: 5′-TCCGTTGGTATCCTCGTTTC-3′) and *Wolbachia* (IS5-F: 5′-CTGAAATTTTAGTACGGGGTAAAG-3′ and IS5-R: 5′- CAAGCATATTCCCTCTTTAAC-3′) sequences. Saliva screening to check the presence of *Wolbachia* was also done via PCR (with IS5 primers) using saliva samples of infected and non-infected mosquitoes. Mosquito sequences in this case were detected with primers for the ribosomal protein gene RpS17 [23].

### Statistical analysis

In all cases, general linear models were employed to examine the effects of the variables age and infection status and their interaction with one another. Models demonstrating significance for the variable infection status were then followed by individual t-tests examining the differences between infected and uninfected mosquitoes for each age class. The proportion of infected and uninfected mosquitoes that obtained blood meals were examined using Mann-Whitney U tests instead of linear models, given the deviation of the data from normality, and were based on four populations of mosquitoes. Chi-square 2×2 contingency tests were employed to examine the relationship between observed behavioral traits and lack of feeding success. The correlation between these traits was quantified using a cox-proportional hazards model for age, with the behavioral traits and lack of blood meal success covariates. All statistical analyses were carried out in STATISTICA v8 (StatSoft, Tulsa, OK).

## Results

### Pre-probing time

**Figure 1:**
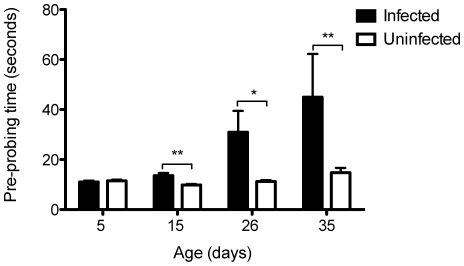
Pre-probing behavior of *A. aegypti* mosquitoes. Comparison of time spent by mosquitoes infected with *Wolbachia* (black bars) or tetracycline treated counterparts (white bars) of different ages (5, 15, 26 and 35 days) after landing on a human hand until the insertion of mouthparts into the skin (*N* = 12–40 per group). Bars depict means±S.E.M. * p<0.05; ** p<0.01 by t-test.

We measured the time mosquitoes spent from first contact with a human volunteer until the insertion of the insect's mouthparts as a measure of pre-probing time. All feeding trials were carried out with individual mosquitoes, which had been starved prior to the assay, at four adult ages (5, 15, 26 and 35-days-old). Mosquitoes that never successfully achieved a blood meal were excluded from this analysis. Overall both age (df = 3, *F* = 13.73, *P*<0.0001) and infection status (df = 1, *F* = 23.18, *P*<0.0001) had a significant effect on the length of pre-probing time. On average infected mosquitoes spend more time pre-probing especially as they age (Fig. 1). This change with age is clearly exhibited by a significant interaction between the variables age and infection status (df = 3, *F* = 8.11, *P*<0.0001). At five days of age infected and uninfected mosquitoes do not differ in their pre-probing time (df = 78, *t* = 0.63, *P* = 0.52), which lasted on average 11 seconds. Uninfected mosquitoes maintained the same foraging time as they aged, while *w*MelPop insects exhibited a steady and significant increase (15d: df = 75, *t* = −3.37, *P* = 0.0012; 26d: df = 63, *t* = −4.17, *P* = 0.014; 35d: df = 48, *t* = −2.25, *P* = 0.0034), reaching a mean length of 45 sec by 35 days of age (Fig. 1).

### Probing time

**Figure 2:**
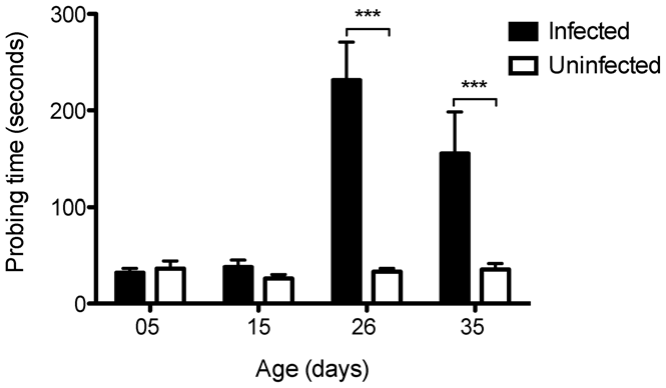
Probing behavior of *A. aegypti* mosquitoes. Comparison of time spent by mosquitoes infected with *Wolbachia* (black bars) or tetracycline treated counterparts (white bars) of different ages (5, 15, 26 and 35 days) from the insertion of mouthparts into the skin of a human hand and the first sign of blood within the insect midgut. (*N* = 12–40 per group). Bars depict means±S.E.M. *** p<0.0001 by t-test.

In the same feeding trials described above, the length of time between insertion of mouthparts and the first visible sign of blood in the abdominal pleura [21] was recorded as probing time for the mosquitoes. As with pre-probing time, the variables of age (df = 3, *F* = 11.36, *P*<0.0001), infection status (df = 1, *F* = 29.46, *P*<0.0001) and the interaction (df = 3, *F* = 10.56, *P*<0.0001) between these two variables were highly significant. Infected and uninfected mosquitoes did not differ in their probing time (∼33 sec) at 5 (df = 78, *t* = −0.46, *P* = 0.64) and 15 (df = 75, *t* = 1.43, *P* = 0.15) days of age (Fig. 2). In contrast, infected mosquitoes at 26 (df = 63, *t* = −3.76, *P*<0.001) and 35 (df = 48, *t* = −4.06, *P*<0.001) days of age took significantly longer during probing, exhibiting up to a seven-fold increase in their probing time relative to uninfected mosquitoes (Fig. 2).

### Blood meal acquisition

**Figure 3:**
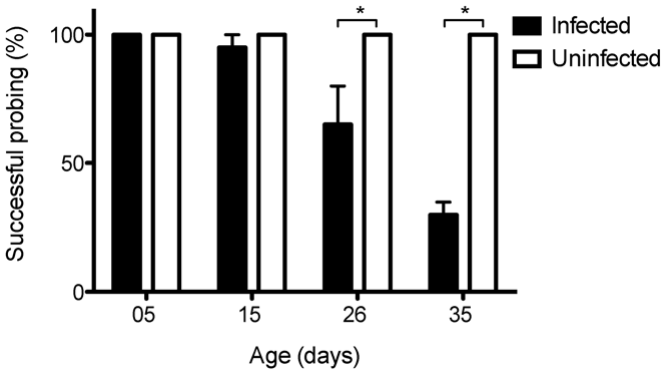
Percent of *A. aegypti* mosquitoes that obtained a blood meal. Percentage of *w*MelPop-infected (black bars) and tetracycline-treated mosquitoes (white bars), that successfully imbibed blood within 10 minutes of observation, by age class. Bars depict medians±quartiles for four replicate experiments, each based on 10 individual mosquitoes. * p<0.05 by Mann-Whitney U test.

In the assays detailed above we then compared the ability of infected and uninfected mosquitoes to obtain blood meals (Fig. 3) using Mann-Whitney U tests. At 5 (*Z* = 0, *P* = 1) and 15 (*Z* = 0, *P* = 1) days of age infected and uninfected mosquitoes did not differ in their success. At 26 (Z = −2.39, P = 0.020) and 35 (Z = −2.39, P = 0.020) days of age infected mosquitoes were less successful at obtaining blood meals in comparison to their uninfected counterparts. Only *Wolbachia* infected mosquitoes refused to land on the hand during the assay or landed but did not take a blood meal. Percentages of individuals that did not land on the human were: 2.5% at 15 d; 0% at 26 d and 7.5% at 35d. Percentages that landed but that did not feed were 5% at 15 d; 37.5% at 26d and 67.5% at 35 d.

### Number of probings

**Figure 4:**
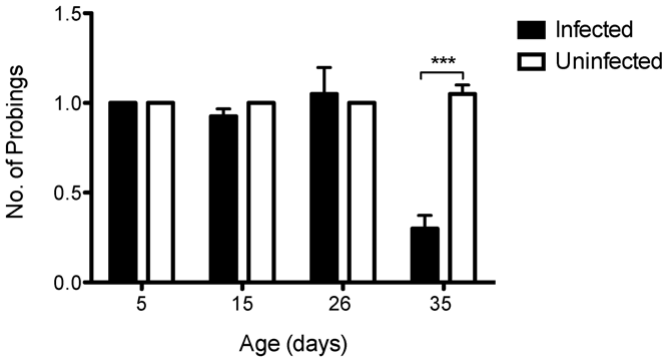
Number of probings in *A. aegypti* mosquitoes. Comparison of number of probings of mosquitoes infected with *Wolbachia* (black bars) or tetracycline treated counterparts (white bars) of different ages (5, 15, 26 and 35 days). (*N* = 40 per group). Bars depict means±S.E.M. *** p<0.0001 by t-test.

It is important to note that as infected mosquitoes aged, the frequency of events where they pierced the skin did not increase despite failed attempts at feeding (Fig. 4). A general linear model revealed that age (df = 3, *F* = 20.47, *P*<0.0001), infection (df = 3, *F* = 29.12, *P*<0.0001) and age X infection (df = 3, *F* = 27.18, *P*<0.0001) were significant determinants of the number of probings. Subsequent t-tests comparing the number of probings between infected and uninfected mosquitoes at each of the age points (data not shown), however, demonstrated that only 35 day old (df = 1, *t* = −8.44, *P*<0.0001) mosquitoes differed. In this case, uninfected females probed more on average per session (1.05±0.05) than *w*MelPop infected mosquitoes (0.3±0.073). This is due to other behaviors, which impaired the infected mosquitoes to feed (see below).

### Additional behavioral phenotypes

**Figure 5:**
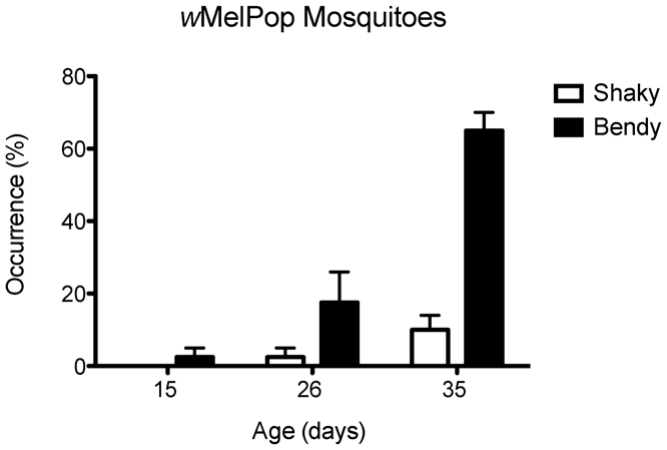
Additional phenotypes observed in *Wolbachia*-infected *A. aegypti*. Proportion of *w*MelPop-infected mosquitoes exhibiting abnormal pre-probing behavior as: body jittering (“shaky”) or bended proboscis (“bendy”) in mosquitoes from their first occurrence at 15 days of age. Bars depict medians±quartiles for four replicate experiments, each based on 10 individual mosquitoes. Neither of these behaviors was observed in *Wolbachia* non-infected mosquitoes.

We recently have reported the appearance of a “bendy” proboscis in association with *w*MelPop, which was the inability of the mosquito to properly orient its mouthparts and insert the stylet into the skin [10]. Here we quantified the occurrence of this trait. The bendy proboscis was never observed in any of the uninfected mosquitoes in this study, nor was it present in a small cohort of very old mosquitoes (∼90 days) we examined in a second pilot study. The phenotype was also not present in 5 day-old infected mosquitoes. The trait first appeared at a low level (2.5%) in 15 day-old *w*MelPop infected mosquitoes and rose to a frequency of 65% by 35 days of age (Fig. 5).

Another phenotype observed, although in lower frequencies, was the jittering action of the insect body (named here as “shaky”) when the mosquito was sitting on top of the human hand (Fig. 5, Video S1). The association between each of these traits and lack of success in blood meal acquisition was explored using 2×2 contingency tests in each of the age classes where the trait was expressed. There was a significant association between the failure to obtain a blood meal and both the bendy phenotype (26d: df = 1, χ^2^ = 14.1, *P* = 0.0002; 35d: df = 1, χ^2^ = 11.8, *P* = 0.0006) and the shaky phenotype (35d: df = 1, χ^2^ = 4.2, *P* = 0.038). Using survival analysis we obtained estimates of the correlation between lack of feeding success and the bendy phenotype (0.63) and the shaky phenotype (0.19). These correlations reveal the presence of a relationship between the traits and success in feeding, but do not completely explain lack of success. There are mosquitoes in the older age classes that fail to feed and that are not shaky or bendy. To discard any possibility that this other abnormal phenotypes were due to the lack of blood feeding, which could have physiologically compromised the mosquitoes we also blood fed females of both groups when they were 3 to 5-days-old and then after 38 days evaluated their feeding behavior. None of the *w*MelPop mosquitoes were able to feed and all presented the bendy proboscis, although all the tetracycline-treated mosquitoes successfully imbibed blood (data not shown).

### Saliva volume and apyrase activity

**Figure 6:**
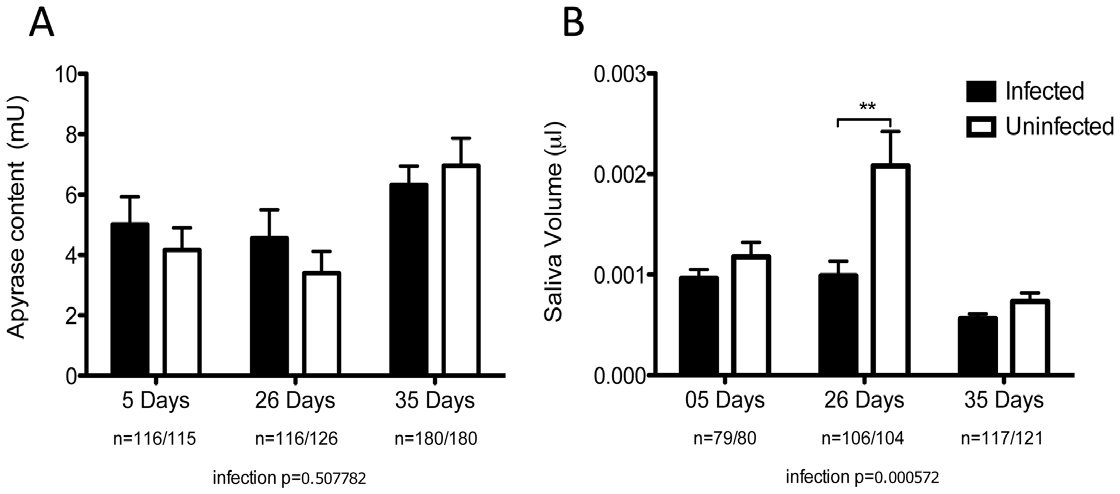
Apyrase content and saliva volume. Comparisons of apyrase and saliva volume of mosquitoes infected with *Wolbachia* (black bars) or tetracycline treated counterparts (white bars) of different ages (5, 26 and 35 days). A) Apyrase activity measured through the release of inorganic phosphate from ATP. B) Saliva volume measured through the sphere volume of saliva droplets. Number of replicates in each group and age are represented. Bars depict means±S.E.M. *P* values relate to univariate tests of significance derived from general linear models. ** indicates *P*<0.01 from t-tests for the specific age category.

To check whether the probing behavior and the additional phenotypes we observed were due to differences in saliva volume and salivary gland apyrase activity we measured both traits in infected and uninfected mosquitoes at three adult ages. Apyrase activity (Fig. 6A) did not differ in infected and uninfected mosquitoes regardless of age (df = 1, *F* = 0.44, *P* = 0.51). Infection status (df = 1, *F* = 11.99, *P*<0.01) and age (df = 2, F = 14.54, *P*<0.0001), however, were determinants of saliva volume (Fig. 6B) and on average infected mosquitoes produced less saliva. When saliva volumes of infected and uninfected mosquitoes were compared to each other for each age class, only the 26 days old mosquitoes were significantly different (df = 1, t = −2.9, *P*<0.01). During collection, 80–100% of the mosquitoes produced saliva droplets. Infection status was not a predictor of whether saliva was produced or not (data not shown).

### Evidence of *Wolbachia* in the saliva

To begin to dissect the functional role of *Wolbachia* in these feeding effects we sought to determine the presence of the bacterium in both the saliva and salivary glands. PCR amplification of the *Wolbachia* wsp gene or mosquito apyrase has shown only the presence of *Wolbachia* in salivary glands, but not in saliva (see Figure S1). The transposable element IS5, present in at least 13 copies within the bacteria genome [24], was also used in extra samples as a very sensitive PCR target (*N* = 16 of each group) but no amplification was obtained (data not shown). These results are supported by the size of the intracellular *Wolbachia* (around 1µm in diameter) [4] and the diameter of mosquito salivary ducts (also about 1 µm) [25], which indicate that even if *Wolbachia* was able to be present in the secreted salivary fluid it would be unlikely to be able to freely move through the salivary ducts.

## Discussion

The results presented here indicate that infection with *w*MelPop alters the blood-feeding behavior of female *A. aegypti* mosquitoes in a manner that intensifies with increasing mosquito age. Older infected mosquitoes spend more time pre-probing and probing, commonly exhibit evidence of shaking and a bendy proboscis, produce less saliva and overall are less successful in obtaining blood meals. None of our experiments revealed any evidence of *Wolbachia* in the saliva or *Wolbachia*-associated changes in salivary apyrase activity.

Female mosquitoes must balance the feeding time required to obtain a sufficient blood meal against the risk of death from human defensive behavior upon their detection [26]. On the other hand, parasites or viruses that are transmitted through the insect saliva benefit from feeding times that enhance transmission but possibly cause a higher risk for the mosquito. Parasite manipulation of insect feeding behavior often includes increases in the insect's probing time or number of probings. This has been seen in mosquitoes infected with malaria parasites [27] and viruses [28], and in trypanosome-infected triatomine bugs [29]. As *Wolbachia* is not transmitted via saliva, the increased length of time infected mosquitoes are taking to feed could be advantageous only if blood-meal sizes acquired were greater and subsequent fecundity rates higher. Our group recently demonstrated evidence to the contrary, with infected females taking smaller blood meals on average [10]. This in conjunction with the data reported here, with overall reduced rates of feeding in combination with physiological changes like the observed “bendy” and “shaky” traits, suggest that the feeding biology is not likely to be a *Wolbachia* parasite “adaptation.”

In the absence of evidence for a specific *Wolbachia* manipulation of the host, blood-feeding effects are likely explained as a by-product of infection or as the direct result of pathogenesis on host cells or tissues. *Wolbachia* are found in many different insect tissues, especially in nervous tissue, muscles and reproductive systems [9],[30]. While in *Drosophila* the deleterious effects of *w*MelPop are related to age and bacterial density [4], the relationship between bacterial density and pathogenicity has yet to be empirically determined for *w*MelPop-infected *A. aegypti*. The decreasing ability to feed with increasing age in combination with the increased prevalence of shaking behavior and the bendy proboscis could be explained by direct damage on host cells or tissues, resulting from *Wolbachia* over-replication. Interestingly, failure to obtain a blood meal cannot be completely explained by these two traits. Alternatively, *Wolbachia* infection may be having direct and targeted effects on neuro-regulators, which would influence their ability to forage for blood and actually feed. The bendy proboscis and shaky phenotypes may represent very extreme endpoints in the process.

The *w*MelPop strain of *Wolbachia* was initially targeted for biocontrol development given its life-shortening capacity [7]. More recently, the presence of *w*MelPop has been shown to block the accumulation of dengue virus in *A. aegypti* [9]. The pattern of the reduced blood-feeding success seen here, like life-shortening leaves the young breeding individuals unaffected while targeting older members of the population. While the dengue transmission rates of *w*MelPop-infected mosquitoes are yet to be estimated, initial concerns that infected mosquitoes, failing to successfully feed, might actually bite more and hence possibly enhance viral transmission appear unfounded. While probing frequency decreased with age in *Wolbachia* infected individuals probing time increased. This was compensated by a reduction in saliva produced by *Wolbachia* infected individuals, which should counteract any potential increases to pathogen transmission risk in *Wolbachia* infected individuals. In conclusion, the presence of *Wolbachia* bacterium in *A. aegypti* mosquitoes significantly reduces their feeding success in an age dependent manner that is more likely a by-product of virulence than a direct manipulation of host behavior. These effects on feeding could substantially improve the efficacy of *w*MelPop biocontrol strategies in combination with the traits of life-shortening and viral blocking.

## Supporting Information

Alternative Language Abstract S1 Translation of the abstract into Portuguese by LAM.
(0.03 MB DOC)

Figure S1 PCR analysis to detect *Wolbachia* in mosquito saliva. Mosquito (apyrase) or *Wolbachia* (WSP) specific primers in infected (InfMq) or uninfected mosquitoes (UnMq), saliva (InfSal or UnSal) or salivary glands (InfSG or UnSG). Specific bands were only detected in whole mosquitoes or salivary glands. Neg = negative control; M = 100bp NEB DNA ladder.
(1.56 MB TIF)

Video S1 Video of jittering behavior (Shaky) seen in *w*MelPop infected mosquitoes only, aged 35 days.
(6.89 MB MOV)
