# Supplementary material for: Human Probing Behavior of Aedes aegypti when Infected with a Life-Shortening Strain of Wolbachia
Source: PLoS Negl Trop Dis. 2009 Dec 15;3(12):e568. doi: 10.1371/journal.pntd.0000568 (PMC2788697; doi:10.1371/journal.pntd.0000568)
Supplement: Alternative Language Abstract S1 — Translation of the abstract into Portuguese by LAM. (0.03 MB DOC) [file pntd.0000568.s001.doc]

Comportamento de alimentação de *Aedes aegypti* em humanos, quando infectados com uma cepa de *Wolbachia* que diminui a longevidade

**Princípio**

Mosquitos são vetores de importantes patógenos em países tropicais e sub-tropicais. As estratégias de controle atuais se baseiam quase na totalidade em inseticidas, aumentado problemas relacionados ao alto custo, ocorrência de resistência e efeitos adversos a organismos não-alvo. Estratégias alternativas incluem a proposta de uso de agentes que causam redução na longevidade dos mosquitos, como a bactéria *Wolbachia*. Ao reduzirmos a longevidade do vetor, a *Wolbachia* poderá potencialmente reduzir a capacidade vetorial de populações de mosquitos. Recentemente conseguimos transifectar estavelmente mosquitos *Aedes aegypti* com uma cepa desta bactéria que causa redução de longevidade, a *w*MelPop. Estamos estudando vários aspectos de sua interação com o mosquito para determinar o impacto que poderá causar na transmissão de patógenos bem como a habilidade de invasão em populações de *A. aegypti*.

**Metodologia/ Principais Resultados**

Neste estudo examinamos o comportamento de alimentação sanguínea de mosquitos infectados com *Wolbachia* para tentarmos entender o impacto da infecção da *Wolbachia* na biologia do mosquito e em particular na capacidade vetorial. O comportamento de mosquitos infectados com *w*MelPop de 4 idades diferentes foi estudado e comparado com mosquitos não-infectados após gravação de vídeos em experimentos de alimentação em mão humana. Insetos positivos para *Wolbachia*, a partir de 15 dias de idade, mostraram um aumento drástico no tempo que levaram na busca e na alimentação sanguínea em comparação com não-infectados. Outras duas importantes características para a alimentação sanguínea, o volume de saliva e o conteúdo da enzima apirase, foram também estudados.

**Conclusões/ Importância**

Quando mosquitos *A. aegypti* infectados com *w*MelPop ficam mais velhos, eles mostram dificuldade para completarem, com sucesso e eficiência, o processo de alimentação sanguínea. Mosquitos infectados com *Wolbachia*, em média, produzem volumes menores de saliva mas que contém a mesma quantidade de atividade de apirase quando comparados com mosquitos não infectados. Estes efeitos no comportamento de alimentação sanguínea poderão reduzir a capacidade vetorial dos mosquitos e indicam mudanças fisiológicas em fêmeas infectadas com *Wolbachia*.
