# Supplementary figures and images for: Human Probing Behavior of Aedes aegypti when Infected with a Life-Shortening Strain of Wolbachia
Source: PLoS Negl Trop Dis. 2009 Dec 15;3(12):e568. doi: 10.1371/journal.pntd.0000568 (PMC2788697; doi:10.1371/journal.pntd.0000568)

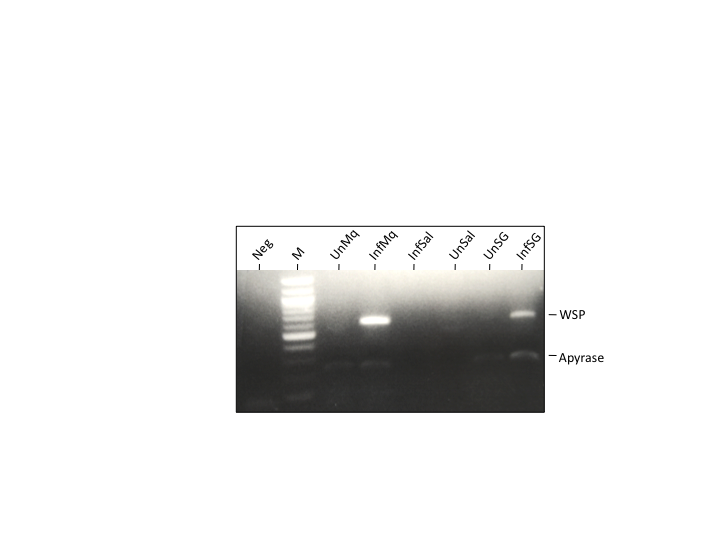

Supplement: Figure S1 — PCR analysis to detect Wolbachia in mosquito saliva. Mosquito (apyrase) or Wolbachia (WSP) specific primers in infected (InfMq) or uninfected mosquitoes (UnMq), saliva (InfSal or UnSal) or salivary glands (InfSG or UnSG). Specific bands were only detected in whole mosquitoes or salivary glands. Neg = negative control; M = 100bp NEB DNA ladder. (1.56 MB TIF) [file pntd.0000568.s002.tif]
